# Supplementary material for: Hyponatraemia reversibly affects human myometrial contractility. An in vitro pilot study
Source: PLoS One. 2020 Jan 23;15(1):e0220020. doi: 10.1371/journal.pone.0220020 (PMC6977732; doi:10.1371/journal.pone.0220020)
Supplement: S1 File — (DOCX) [file pone.0220020.s001.docx]

Oxytocin added in second part of the study.

Our methods are described in detail in the manuscript, with the exception of the method of dilution described below.

In the second part of the study OT was added to the 5 ml organ baths using the following methods for dilution:

500µL av 5 IE +2000µL Tyrode = **1 IE**  100µl

100µL av 1 IE + 900µL tyrode =**0,1 IE**. 100µL

100µL av 0,1  IE + 900µL tyrode =**0,01 IE**. 100µL
