# Supplementary material for: Hyponatraemia reversibly affects human myometrial contractility. An in vitro pilot study
Source: PLoS One. 2020 Jan 23;15(1):e0220020. doi: 10.1371/journal.pone.0220020 (PMC6977732; doi:10.1371/journal.pone.0220020)
Supplement: Attachment — Submitted filename: review Moen Brudin 2019.pdf [file pone.0220020.s006.pdf]

120 mM Na 1 hour then 136 mM 1 hour; repeat

Prior paper 2009 looked at oxytocin in multvar regression, but using 5 U total dose and bimodal analysis, showed .072 p value, although CI - .9 – 7.4. Thus**, probably should not strongly say that oxytocin is not related to hyponatremia, as noted in this abstract**. Not to review a 2009 publication, but excessive oxytocin may cause low Na in some patients.

Also no mention of inappropriate ADH due to labor in either paper

Phase plot reference 8 should be 10, page 14. The phase plot analysis proposed by the Warwick group actually was not intended primarily as a measure of effectiveness of contractions (especially in vivo), rather normalizing results among different samples.

While customary in the field of uterine contractility, the area under the curve (AUC) is actually a force-time integration, which is a mechanical impulse. This unfortunately was also chosen decades ago as the word to describe an action potential, hence the potential (pun intended) confusion in a field where the tissue emits both an impulse in the form of an action potential and an impulse in the form of an integrated force. Just a comment, not a recommendation for change, since that boat left dock a while ago. If would be nice to occasionally see the correct terms used, however.

Intro –

- S**hould mention oxytocin at least as a possibility for causing hyponatremia**
- Cl- is largely distributed by passive electrical properties – donnan/Nernst
- Intro describing the action potential is a bit long and the main point is only that these ions create electrical activity that "opens" voltage gated calcium channels which initiates the contraction. If you want to go into detail, you should also mention the Na/Ca exchanger which is probably more important in Na metabolism than the Na channel, esp regarding duration of contractions

Sampling

- 4 years to write manuscript?
- Omit humanitarian or define it. Given the routine nature of this biopsy, we really don't even need to know other than "clinical indications" and implying that none were done for research purposes alone.
- Typo labouratory – "were" to where
- Putting normonatraemic Tyrode's once (earlier) is enough, redundant is redundant. Tyrode's is normal Na. Also, you realize that Mg2+ is a potential tocolytic, probably should comment at some point. Most groups avoid Mg, although for these studies it probably makes no difference. For storage/transport on ice, you should mention pH, though since real Tyrode's is ~ 6.5 unless outgassed with CO2
- **Did you stabilized the 100 mg (9.8 mN) tension after tissue creep (i.e. rest period)?**
- You studied one biopsy at a time, although up to 8 strips simultaneously, correct?

First part of the study

- No need to repeat studying one biopsy at a time
- Phase plot reference is Gullum – 10. My guess is that you need to use endnote or similar program, since it looks like **several of your references are incorrectly numbered**

Second part of the study

- No need to repeat studying one biopsy at a time
- This description of oxytocin concentrations brings into question if the 82.5 nM oxytocin was used

Solutions

- While this is a complete description, it would be good to not repeat all the unchanged chemicals, but simply state the that the S solution was made hyponatremic *and hypoosmotic* by reduction of NaCl to 97.4 mM from 114 mM. Also, if oxytocin was added to the rest period (which I am not sure), you could place oxytocin here as well so that it is clear all tissues were exposed to oxytocin. **On the other hand, if all tissues were not exposed to oxytocin, please correct your description on page 7** (top)

Statistics

- Do you mean "mean values for each biopsy..? – or each tissue strip?

Results

- Exclusion due to technical problems, or simply the tissue strip failed to contract (which can be up to 20% and still be OK)
- Now you have to define humanitarian. I think you mean "elective"
- Earlier you probably should note that none of your subjects were in spontaneous labor

First part

- First sentence is not a sentence
- **Needs to be really clear – *peak* force is the force change from "resting" to maximum force generated. (or however you measured)**
- **Fig 1 is confusing. The 1 h is not clear. How many strips is this? Upper panel is noted, but not lower panel. Needs much better labeling**
- Fig 2. As above, amplitude is probably peak force, should note if measured from onset to peak or simply peak from absolute 0.
- Fig 3 phase plots of the contractions above?
- the really interesting finding is the oscillatory behavior in the lower 4 tracings in the lower panel – if only I could figure out the history of the tissues.

Second part

- **This is confusing to read – could this just be a table?**
- All the figure legends should be put together at the end, near the figures.

Discussion

- This is where the concept of peak force rather than just force helps keep confusion with AUC (which to some is a force) low.
- All changes were reversible in the…. Indicating that hyponatremia or *hypoosmolarity …*
- Probably could mention Parkington's work, but it is a bit overboard to attribute your results to spike-like rather than plateau potentials.
- The paragraph that starts with tachysystole doesn't hang together well. You never really got close to 5 contractions in 10 min, so I would suggest you avoid this topic
- You really short-changed the discussion on the 2nd part of the study. What do you think caused multiphasic appearance? What is the physiological underpinning, and why would OT bring that out?
- Pulsatile OT is neither here nor there -suggest you either justify or omit

- What about multiphasic contractions in vivo – do your multiphasic relate to them (doubling, tripling, etc)
- Given the effort on phase plots – they are also a way to quantify multiphasic behavior – that is the areas of the closed circles of the plots return a measure of the impulse attributable to each phase of the contraction. I actually don't think this must be done because it is a lot of calculating for very little information, but just to point out that it could be done for the 2nd part oxytocin effects.

Streghts (sic-type) and limitations

- **Our studies on excised human tissue may more closely represent the physiology of human labor better than rodent model**. Or similar phrasing
- OT was used to initiate contractions and establish a constant starting point to investigate the specific effects of hyponatremia/ hypoosmolarity on uterine contractions.
- You already mention type II error, no need to have the last two lines as a limitation
- **The key limitation was that you studied hyponatremia without maintaining osmolarity constant, so the effects of Na are mixed with hypoosmolarity**. However, since this is the clinical condition you wished to mimic, that may be what you intended to do.

Conclusion

- Some of the other factors for dystocia could be …. For example Sue Wray's group showed that pH is a key factor as well.
- Fluid intake was a conclusion of your prior paper, so you probably should merely say that low Na is a modifiable clinical parameter

In summary

This is a very good paper with very good data. While the philosophy of PLoS One is to publish data regardless of perceived relevance, this paper has both good data and relevance. As pointed out above, there are a few areas of potential confusion and a few missed opportunities. The authors should consider most of this review as suggestions, with questions requiring answers are written in **bold.**

This manuscript satisfies PLoS One criteria for publication
